# Neuromodulation to Treat Substance Use Disorders in People With Schizophrenia and Other Psychoses: A Systematic Review

**DOI:** 10.3389/fpsyt.2022.793938

**Published:** 2022-02-14

**Authors:** Samantha Johnstone, Maryam Sorkhou, Nada Al-Saghir, Darby J. E. Lowe, Vaughn R. Steele, Godfrey D. Pearlson, David J. Castle, Tony P. George

**Affiliations:** ^1^Addictions Division, Department of Psychiatry, Centre for Complex Interventions, Centre for Addiction and Mental Health, University of Toronto, Toronto, ON, Canada; ^2^Olin Center for Neuropsychiatric Research, Institute of Living, Hartford, CT, United States; ^3^Department of Psychiatry, Yale School of Medicine, New Haven, CT, United States

**Keywords:** substance use disorder, nicotine, cannabis, psychosis, schizophrenia, neuromodulation, rTMS, tDCS

## Abstract

**Background:**

Substance use disorders (SUDs) are a common yet poorly studied comorbidity in individuals with psychotic disorders. The co-occurrence of the two complicates recovery and interferes with pharmacological and behavioral treatment response and adherence. Recently, researchers have been exploring both invasive and non-invasive neuromodulation techniques as potential treatment methods for SUDs. We review the evidence that neuromodulation may reduce substance craving and consumption in individuals with schizophrenia.

**Methods:**

A comprehensive literature search of PubMed, MEDLINE, and PsycINFO databases was conducted (*N* = 1,432). Of these, we identified seven studies examining the effects of repetitive transcranial magnetic stimulation (rTMS) and two studies using transcranial direct current stimulation (tDCS) on drug consumption and craving in schizophrenia or schizoaffective disorders.

**Results:**

Despite the limited number of studies in this area, the evidence suggests that rTMS to the dorsolateral prefrontal cortex (DLPFC) may reduce cannabis and tobacco use in patients with schizophrenia and schizoaffective disorder. Findings with tDCS, however, were inconclusive.

**Discussion:**

Our systematic review suggests that rTMS applied to DLPFC is a safe and promising therapeutic technique for the management of comorbid schizophrenia and SUDs, with the majority of the evidence in tobacco use disorder. However, there was substantial heterogeneity in study methods, underscoring the need to optimize stimulation parameters (e.g., frequency, duration, and target regions). Larger clinical trials are needed to establish the efficacy of rTMS in reducing drug consumption and craving in psychotic patients, ideally in comparison to existing pharmacological and behavioral interventions.

## Introduction

Schizophrenia (SCZ) is a serious mental illness affecting nearly 20 million people worldwide (1). SCZ is characterized by positive (i.e., paranoia and hallucinations), negative (i.e., amotivation and anhedonia), disorganized (i.e., thought disorder and disorganized behaviors), and cognitive (i.e., deficits in attention and sensory processing) symptoms (2). The course and prognosis of SCZ is often complicated by co-occurring substance use disorders (SUD), evidenced by a global prevalence of ~42% for any SUD, including illicit drugs (27.5%), cannabis (26.2%), and alcohol [24.3%; (3)]. Such high levels of comorbidity are potentially due to, inter alia, shared genetic and environmental factors increasing SUD vulnerability [for review see (4, 5)] or to alleviate cognitive and psychotic symptoms (6). Use of psychoactive substances can interfere with antipsychotic medication (7), are associated with reduced adherence to SCZ interventions (8), and can lead to symptom exacerbation (9). There are mixed findings with respect to antipsychotics for treating SUDs in SCZ and preliminary support for the use of naltrexone in reducing alcohol use in SCZ (10). Behavioral interventions have also shown some success in reducing substance use, mostly during the intervention period (10). However, research remains relatively limited as individuals with co-occurring serious mental illnesses are often excluded from SUD clinical trials. Moreover, these methods are difficult to implement in SCZ patients; psychotic symptoms and cognitive deficits may reduce patients' ability to engage meaningfully in SUD behavioral interventions (11), while certain pharmacological addiction interventions may worsen positive symptoms of psychosis (12–14), urging investigation into novel and effective interventions for SCZ patients.

Invasive and non-invasive neurostimulation techniques are emerging innovative treatments that have been investigated in the context of treatment-resistant illnesses (15) in individuals who struggle with adherence. As such, they are a promising modality for treating SUDs in SCZ as they can directly target putative brain regions, such as the prefrontal cortex and nucleus accumbens, that are associated with SCZ and SUD pathophysiology (4) with less effort than is required for medication compliance. Moreover, they are safe, time-effective, and patient-friendly, offering a neuroscience-based treatment that may be superior to conventional medications and behavioral therapies. Such techniques include non-invasive repetitive transcranial magnetic stimulation (rTMS), transcranial direct current stimulation (tDCS), or invasive deep brain stimulation (DBS). The purpose of this review is to systematically review the evidence that neurostimulation techniques reduce substance craving and consumption in individuals with SCZ.

### Neurostimulation Methods

#### Repetitive Transcranial Magnetic Stimulation

rTMS is a non-invasive stimulation method that involves positioning an electromagnetic Figure-of-8 or H-coil on the scalp to produce a time-varying magnetic field (16, 17). This current can be localized to specific brain regions to modulate neurotransmission (18). Options include low- or high-frequency rTMS, which tend to produce inhibitory or excitatory effects, respectively (17). A variation of rTMS is intermittent or continuous theta burst stimulation, which generally produces similar results, usually with shorter session duration (19, 20). rTMS is well tolerated, with some people reporting headache, tingling, or lightheadedness (20–22) and rarely cognitive deficits or seizures [0.071%; (23, 24)], with H-coil carrying a slightly higher risk than Figure-of-8.

#### Transcranial Direct Current Stimulation

tDCS is another non-invasive stimulation method that involves a low-intensity, steady-state direct current that is delivered to a localized brain region via two or more electrodes on the scalp (18, 25). There are variations between protocols regarding the size and number of electrodes, duration of stimulation, and current strength that modify the dispersion of the current to the brain. tDCS electrodes can either increase (if anodal) or decrease (if cathodal) the likelihood of neuronal firing by modulating the resting membrane potential (25). Furthermore, prolonged stimulation may modify synaptic plasticity via long-term potentiation or depression (17, 25). tDCS is relatively low risk, with some patients reporting sleepiness, minor discomfort, or mild burning or pain in the neck or scalp (26).

#### Deep Brain Stimulation

DBS is an invasive technique, where microelectrodes are embedded in the brain, thus allowing for sustained modulation of neuronal firing to regulate neurotransmission in specific brain regions (18). Embedded electrodes are coupled with a pulse generator to facilitate continuous stimulation (18). Although DBS offers a more localized and deeper signal that can modulate oscillatory activity, it involves a surgical procedure and thus carries risks including infection or hemorrhage (27).

### Evidence for Neurostimulation in Substance Use Disorders

The evidence to date suggests that stimulation of regions in the mesocorticolimbic system may modulate dysregulated neurotransmitter release, thus reducing craving and consumption of addictive substances (16, 28). The most consistent positive results occur when multiple sessions of high-frequency (>10 Hz) stimulation is applied to the dorsolateral prefrontal cortex (DLPFC), as this enhances its inhibitory actions on the mesolimbic DA circuits (16, 18, 28). Indeed, preliminary studies using small sample sizes have found that after 10- 20 sessions, both Figure-of-8 and H-coil rTMS are effective at reducing alcohol cravings (29–32) and consumption (33). Furthermore, figure-of-8 coil rTMS has been effective in reducing cigarette craving and consumption (34–37), cocaine craving (38, 39) and producing greater abstinence rates (40, 41) when applied to the DLPFC. tDCS has also shown promising results in reducing craving and consumption of alcohol, opioids, cannabis, cocaine, and methamphetamines [for review see (18)], and tobacco (42). Additionally, several case series investigating DBS targeting the nucleus accumbens suggest it may also be effective in reducing alcohol craving and intake as well as cocaine use [for review see (17)].

However, there are some studies that are inconsistent with the above findings (43–45). Such discrepancies are likely to due to inconsistencies in stimulation parameters, such as number of pulses, duration of stimulation, stimulation frequency, and number of sessions. These parameters influence whether stimulation is excitatory, the magnitude of the electric field delivered, neuronal activation, and tolerability (23). As such, investigation into the effectiveness of neurostimulation in treating SUDs is paramount, as is standardization of stimulation parameters and extension of this research to individuals with comorbid SCZ and SUDs.

### Neurostimulation in Comorbid Schizophrenia and Substance Use Disorders

Given the high prevalence of SUDs (3) coupled with the lack of effective treatments for addiction in SCZ patients, novel, low-effort, and quick to administer treatments are needed. The neuropathological correlates of SCZ, including dysregulated dopaminergic, serotonergic, and cholinergic systems result in characteristic psychopathological symptoms, along with deficits in reward processing and cognitive function (46). Dysregulated responses to rewarding stimuli are thought to underlie the increased reinforcement of substances in SCZ relative to non-psychiatric controls [for review see (5)]. Moreover, individuals with SCZ may use substances as a way to cope with negative symptoms (e.g., restricted affect) and/or attenuate cognitive deficits (9, 47). In light of the promising effects of neurostimulation on regulation of neurotransmitters (28), improvements on negative symptoms in SCZ (48) that may contribute to use, improvements on depressive symptoms (49), cognitive functioning, and reductions in cravings and consumption (17, 18) in non-psychiatric SUDs, individuals with SCZ stand to benefit from investigation into the utility of neurostimulation.

Accordingly, we review the available evidence on neurostimulation techniques as a treatment modality for SUDs in SCZ. In an effort to be comprehensive, all psychotic-spectrum disorders were included in the search, however, only studies assessing SCZ and schizoaffective disorder (SCA) were found.

## Methods

### Search Strategy

A comprehensive literature search was conducted by two of the authors (SJ and MS) using PsycINFO, PubMed, and MEDLINE following PRISMA guidelines. Search terms included: neuromodulation, neurostimulation, stimulation, (repetitive) transcranial magnetic stimulation, transcranial direct current stimulation, theta burst stimulation, deep brain stimulation, vagus nerve stimulation, and psychosis, schizophrenia, schizoaffective disorder, schizotypal, delusional, schizophreniform, psychotic, bipolar psychosis, depressive psychosis, and substance use disorder, substances, addiction, drugs, cocaine, crack, cannabis, tobacco, nicotine, alcohol, methamphetamines, amphetamines, opioids. We included only randomized sham-controlled trials (RCT), open-label studies, or case studies whose population had a psychosis-spectrum disorder and whose primary or secondary outcomes were an end point measure of substance consumption or craving. Exclusion criteria included substance-induced psychosis, non-validated measures of substance use, and reviews or meta-analyses.

### Risk of Bias

Risk of bias was evaluated using the Cochrane Collaboration Tool (50), which assesses studies on the following criteria: random sequence generation, allocation concealment, blinding of participants and research personnel, blinding of outcome measures, incomplete outcome data, and selective reporting of results.

## Results

### Study Characteristics

**Figure 1:**
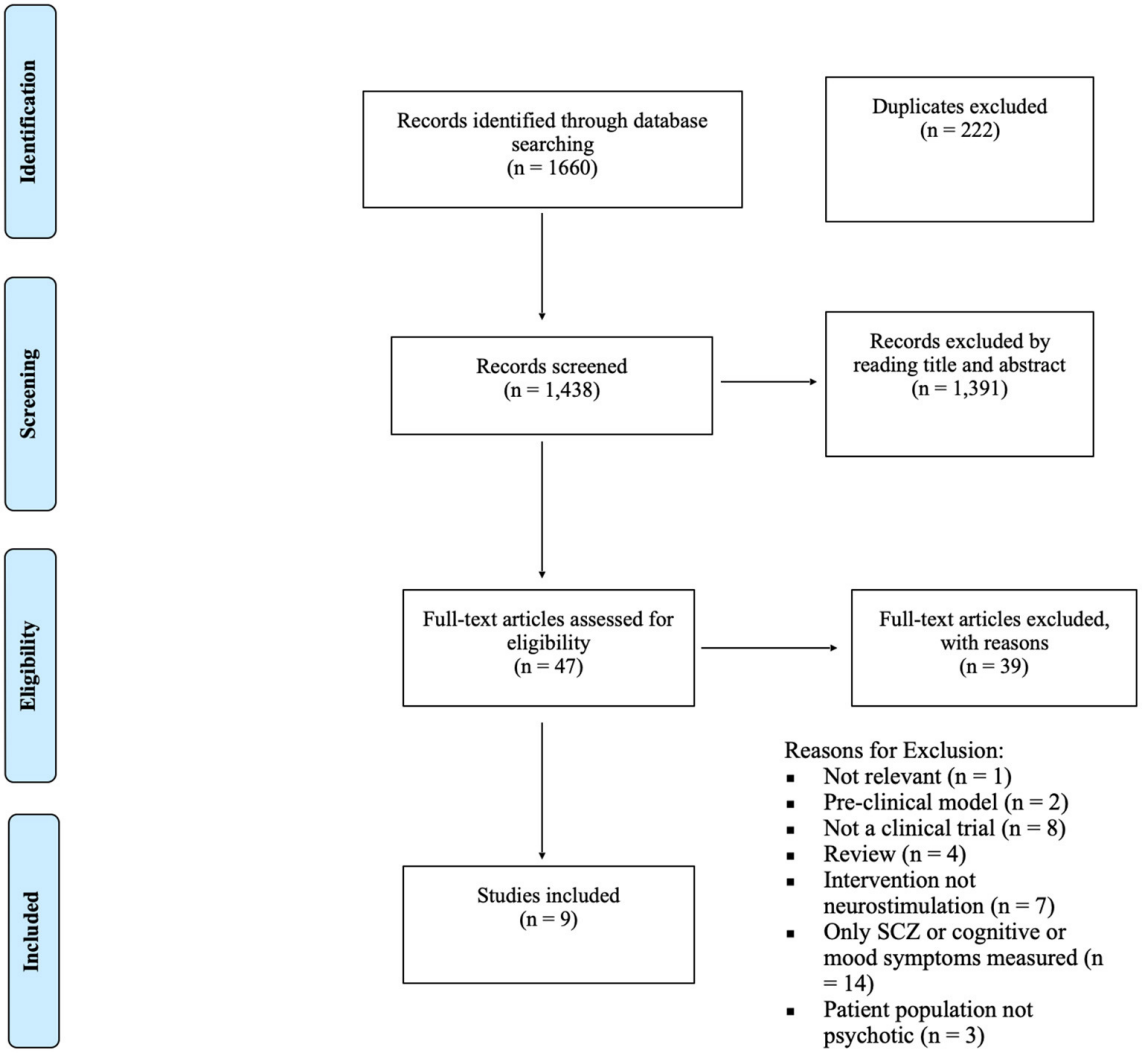
PRISM-A diagram depicting study inclusion process (51).

As depicted in Figure 1, after identifying 1,438 unique studies, 47 studies were assessed for full eligibility, leaving eight published papers and one unpublished manuscript (rTMS = 7, *n* = 204; tDCS = 2, *n* = 49). Seven of the included studies were RCTs whereas the other two employed an open-label design. All but one of the studies examined the effects of neurostimulation on cigarette craving or consumption in individuals with SCZ or SCA. The remaining study investigated cannabis craving and consumption along with cigarette consumption in SCZ. The main characteristics of the included studies are described in Table 1 below.

**Table 1:** Outcomes of cochrane risk of bias assessment.

	**Random sequence generation (selection bias)**	**Allocation concealment (selection bias)**	**Blinding of participants and personnel (performance bias)**	**Blinding of outcome assessment (detection bias)**	**Incomplete outcome data (attrition bias)**	**Selective reporting (reporting bias)**
Brunelin et al. (52)	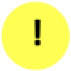	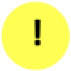	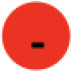	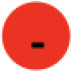	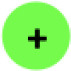	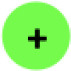
Huang et al. (53)	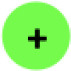	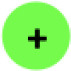	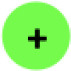	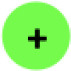	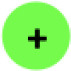	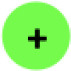
Kamp et al. (54)	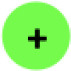	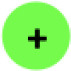	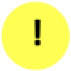	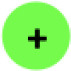	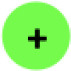	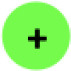
Kozak et al. (55)	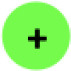	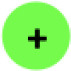	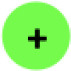	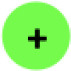	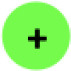	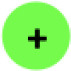
Kozak-Bidzinski et al. (56)	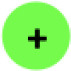	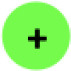	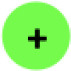	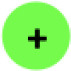	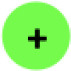	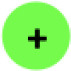
Prikryl et al. (57)	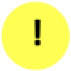	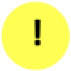	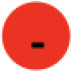	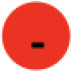	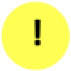	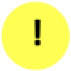
Prikryl et al. (58)	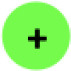	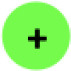	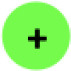	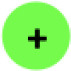	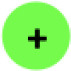	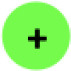
Smith et al. (59)	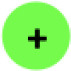	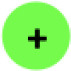	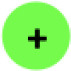	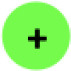	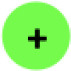	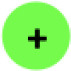
Wing et al. (60)	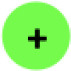	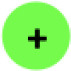	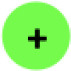	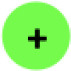	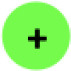	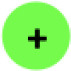

*Green, Low risk of bias; Yellow, Medium risk of bias; Red, High risk of bias*.

### Risk of Bias Assessment

Table 1 shows the results of the risk of bias assessments. Overall, the seven RCTs were of high methodological quality with all but one scoring a 6, while the two open-label studies indicated a high risk of bias.

### RTMS Studies

#### Craving

As seen in Table 2, two studies (55, 60) investigated the effects of rTMS on cigarette cravings, measured by the Tiffany Questionnaire for Smoking Urges (TQSU). While both studies administered rTMS at 20 Hz to the DLPFC bilaterally, the short-term (6 sessions; 3 days) study found no reduction in cigarette cravings or withdrawal (55) at the end of stimulation, whereas the longer intervention (20 sessions; 28 days) found a significant reduction in desire and intention to smoke cigarettes in the active group relative to the sham group (60). However, only the acute trial involved contingent abstinence, potentially resulting in increased cravings for participants, making direct comparison difficult. One study investigated the effect of rTMS on the bilateral DLPFC (20 Hz, 20 sessions) on cannabis cravings and withdrawal (56). While not statistically significant, the active group reported greater (50%) reductions in cravings than the sham group, particularly in terms of expectations of positive outcomes (e.g., feeling more social) after using cannabis.

**Table 2:** Main findings from repetitive transcranial magnetic stimulation studies.

**References**	**Study design**	**Sample**	**Stimulation target**	**Stimulation frequency (Hz)**	**Number of sessions**	**Primary SUD outcome (effect size)**	**Secondary outcomes (effect size)**	**summary of relevant results**
Huang et al. (53)	Randomized, double blind, parallel, sham-controlled Active = figure-8 Sham = identical coil shape produces sound but no stimulation	SCZ (*n* = 37, active = 19), M/F = 37/0	Left DLPFC	10	21	Tobacco use disorder; cigarettes smoked from baseline to 21 day follow up (active *d =* 2.06, *p* < 0.05; control *d =* 0.2, *p* = 0.18; difference *f* = 0.98, *p* < 0.001)	PANSS, Wisconsin Card Sorting Test, MADRS (ns)	Active group showed a statistically significant reduction in number of cigarettes smoked compared to control group. No statistically significant differences in secondary measures after treatment, smoking not related to secondary measures.
Kamp et al. (54)	Double blind, randomized, parallel, sham-controlled Active = figure-8, Sham = distortion of coil 45° away from skull	SCZ (*n* = 67, active = 32), M/F = 55/12	Left DLPFC	10	15	Tobacco use disorder; cigarettes smoked from baseline to 21 day follow up (*f* = 0.08, *p* = 0.54). Correlation between number of cigarettes smoked and reduction (*r*_23_ = 0.385, *p* = 0.057)	Covariates: PANSS positive symptoms, gender, mood stabilizers, benzodiazepines (ns), antidepressants (*f* = 0.42, *p <* 0.01)	rTMS did not significantly reduce the number of cigarettes smoked. Higher number of cigarettes smoked tended to predict a greater reduction.
Kozak et al. (55)	Counter-balanced, double blind, cross-over Active = figure-8 Sham = single wing tilt	SCZ (*n* = 13) HC (*n* = 14)	Bilateral DLPFC	20	6	Tobacco use disorder; MNWS, TQSU: time x diagnosis x rTMS (ns)	SDR (ns), HVLT discrimination, time x rTMS (*f* = 0.45, *p* = 0.016)	Acute administration of rTMS did not reduce abstinence-induced cravings or withdrawal.
Kozak-Bidzinski et al. (56)	Randomized, double blind, parallel, sham-controlled	SCZ (*n* = 19, active = 9), M/F = 18/1	Bilateral DLPFC	20	20	Cannabis use disorder; baseline to 28 day follow up; change across groups Grams/day (*d* = 0.72, *p =* 0.21), NarcoCheck (*d* = 0.55, *p =* 0.26), MCQ (*d* = 0.49, *p* = 0.19) MWC (*d* = −0.22, *p* = 0.58) Tobacco use disorder; cigarettes/day (*d* = 0.96, *p* = 0.01)	PANSS total (d *=* 0.79, *p* = 0.02), CDSS (ns), HVLT, SDR, BART, TMT, digit span, TOL, KDDT, MMN (ns) CPT hit reaction time (*d* = 0.17, *p* = 0.048), variability (*d* = 1.64, *p* = 0.04).	rTMS produced greater reductions of medium magnitude in self-reported and urinalysis cannabis use and cigarettes smoked. Greater reductions in appetitive states of cannabis craving in active group.
Prikryl et al. (57)	Open-label Figure-8	SCZ (*n* = 18), M/F = 18/0	Left DLPFC	10	15	Tobacco use disorder; baseline to 21st day of stimulation; cigarettes/day (*d =* 0.69, *p* < 0.01)	PANSS total (*d =* 1.5, *p <* 0.01) MADRS (*d =* 2.1, *p <* 0.01)	rTMS significantly reduced the number of cigarettes smoked per day during the stimulation period.
Prikryl et al. (58)	Double blind, randomized, parallel, sham-controlled Active = figure-8 Sham = identical coil shape produces sound but no stimulation	SCZ or SCA (*n* = 35, active = 18), M/F = 35/0	Left DLPFC	10	21	Tobacco use disorder; cigarettes smoked from baseline to 21 day follow up (active, *d* = 0.67, *p <* 0.01; control, *d* = −0.03, *p* = 0.59)	PANSS, MADRS, CDSS (ns)	rTMS significantly reduced the number of cigarettes smoked in the active group with no change in the control group. Other psychopathology symptoms not related to changes in smoking.
Wing et al. (60)	Counter-balanced, randomized, double blind, parallel, sham-controlled Active = figure-8 Sham = single wing tilt	SCZ or SCA (*n* = 15, active = 6)	Bilateral DLPFC	20	20	Tobacco use disorder; cravings (TQSU; *p <* 0.05), abstinence (ns)	N/A	rTMS significantly reduced desire and intention to smoke in the active group relative to sham group. There was no effect of rTMS on abstinence.

*ns, not significant, statistics not reported*.

*TQSU, Tiffany Questionnaire on Smoking Urges; MCQ, Marijuana Craving Questionnaire; MNWS, Minnesota Nicotine Withdrawal Scale; MWC, Marijuana Withdrawal Checklist; PANSS, Positive and Negative Syndrome Scale; MDRS, Montgomery Asberg Depression Rating Scale; HVLT, Hopkins Verbal Learning Test; CDSS, Calgary Depression Scale for Schizophrenia; CPT, Continuous Performance Test; TMT, Trail Making Test; BART, Balloon Analog Risk Task; TOL, Tower of London Task; KDDT, Kirby Delay Discounting Test; MMN, Auditory Mismatch Negativity; MCCB, MATRICS Consensus Cognitive Battery*.

#### Consumption

Five of the identified studies investigated the effects of rTMS on cigarette or cannabis consumption. Four of these studies applied 10 Hz (15-20 sessions) to the left DLPFC and assessed changes in cigarette consumption from baseline to the 21st day of stimulation (57) or after a 21 day follow-up (53, 54, 58). In three of the four studies, rTMS significantly reduced the number of cigarettes smoked relative to the control group; one study did not find a significant reduction (54). However, Kamp et al. found that individuals in the active group who smoked a higher number of cigarettes reported a greater reduction in consumption (54). One study investigating the effects of bilateral DLPFC (20 Hz, 20 sessions) found trending reductions in self-reported and biologically verified cannabis use in the active group that were greater than the sham group, as well as a statistically significant and strong reductions in cigarette use (56).

### TDCS Studies

#### Craving

Table 3 depicts the results of the tDCS studies. One study investigated the effect of tDCS on cigarette cravings. Smith et al. (59) applied 2 mA through a cathode to the contralateral supraorbital ridge and through an anode to the left DLPFC (20 min; five sessions) and found no reduction in urge to smoke or dependence, as measured by the Questionnaire on Smoking Urges.

**Table 3:** Main findings from transcranial direct current stimulation studies.

**References**	**Study design**	**Sample**	**Stimulation site**	**Stimulation density**	**Stimulation duration**	**Number of sessions**	**Anode/cathode**	**Primary SUD outcome (effect size)**	**Secondary outcomes (effect size)**	**Summary of relevant results**
Brunelin et al. (52)	Open-label proof of concept	SCZ (*n* = 16), M/F = 6/10	Left temporo-parietal junction Left prefrontal region	2 mA	20 min	10	Cathode Anode	Tobacco use disorder, cigarettes smoked (ns)	Auditory hallucination rating scale (*d* = 0.9, *p* = 0.005)	No effect of tDCS was observed on cigarette consumption. Smoking status reduced clinical efficacy of tDCS on hallucinations.
Smith et al. (59)	Randomized, double blind, parallel, sham-controlled Sham = 2 mA lasting only 40s, electrodes in place for 20 min	SCZ or SCA (*n* = 33, active = 17), M/F = 24/9	Contralateral supraorbital ridge	2 mA	20 minutes	5	Cathode Anode	Tobacco use disorder, cigarettes smoked, breathalyzer CO2 levels, QSU (ns)	PANSS, Haddock Hallucination Scale (ns). MCCB (*d* = 1.03, *p <* 0.01)	tDCS did not reduce urge to smoke or cigarette dependence nor did it improve abstinence or psychopathology. tDCS did improve cognitive performance.

*ns, not significant, statistics not reported*.

*PANSS, Positive and Negative Syndrome Scale; MCCB, MATRICS Consensus Cognitive Battery*.

#### Consumption

Two studies investigated the effects of tDCS on cigarette consumption. Brunelin et al. (52) applied 2 mA through a cathode to the left temporo-parietal junction and through an anode to the left prefrontal region for 20 min (10 sessions) and found no effect on cigarette consumption. Moreover, cigarette consumption was associated with a reduction in the clinical efficacy of tDCS on auditory hallucinations. However, there was no sham group in this study. Similarly, when applying 2 mA for 20 min (five sessions), through a cathode to the contralateral supraorbital ridge and through an anode to the left DLPFC, Smith et al. (59) found no reductions in self-reported or biologically verified cigarette abstinence.

### Secondary Analyses

In three out of the four studies that examined cognitive outcomes, tDCS and rTMS were both effective in improving performance on some measures, including the discrimination index on the Hopkins Verbal Learning Test [HVLT; assesses immediate and delayed recall; (55)], hit reaction time and variability on the Continuous Performance Test [CPT; (56)], and the composite score of the MATRICS Consensus Cognitive Battery (assesses a range of cognitive functioning in SCZ) as well as working memory and attention subscales (59). However, there were a few cognitive tasks on which rTMS had no effect depicted in Table 2. With respect to clinical outcomes, two studies found reductions in total scores of the Positive and Negative Symptom Scale (56, 57) and one found improvements on the Montgomery-Asberg Depression Rating Scale (MADRS) as a result of rTMS. Moreover, one study found improvements on auditory hallucinations after tDCS (52). However, three studies found no effects of rTMS (*n* = 2) on the MADRS or PANSS (53, 58) or tDCS (*n* = 1) on PANSS scores or hallucinations (59).

### Adverse Events

rTMS and tDCS procedures were well-tolerated in the included studies. Some participants reported mild to moderate application site pain, neck pain, headache, or dizziness. All resolved naturally (52, 53, 56, 59, 60). No participants dropped out of the study due to pain from the study device. There were no reports of treatment emergent memory or other cognitive deficits, or seizures.

## Discussion

Our review of the extant literature suggests that rTMS applied to the left or bilateral DLPFC may be effective in reducing craving for and consumption of tobacco and cannabis in individuals with SCZ or SCA. However, evidence did not support the efficacy of tDCS in reducing cigarette craving or consumption, possibly due to the limited number of stimulation sessions employed (5–10) relative to the rTMS studies where 15 or more sessions were performed. While the results of studies in this review provide support for continuing investigation of rTMS as an addiction treatment, there remains a need for more robust clinical trials as well as standardization of stimulation parameters.

Based on calculated effect sizes (Tables 2, 3) the evidence suggests that 10 Hz of rTMS directed at the left DLPFC for at least 20 sessions is effective in reducing cigarettes smoked per day. Moreover, 20 Hz for at least 20 sessions directed at the bilateral DLPFC is effective in reducing cravings for cigarettes, cigarettes smoked per day, and- albeit on the basis of a single study might be effective in reducing cannabis use. Interestingly, high-frequency rTMS (10 Hz or more) applied to the DLPFC for a greater number of sessions is also supported by data from neurostimulation studies in non-psychiatric SUD samples (18, 28). While difficult to compare across diagnoses, the lack of efficacy of tDCS on tobacco craving and consumption does not align with literature in non-psychiatric individuals with SUDs, which did show significant effects after 1-5 sessions (18) with a similar intensity (2 mA) and duration (20 min). It is possible that neurobiological underpinnings of SCZ are not concordant with tDCS stimulation targets or that more sessions are needed to see similar effects. Further investigation is needed for conclusive guidance.

Although the effectiveness of rTMS in reducing tobacco cravings in people with SCZ was variable across reviewed studies, the null findings in Kozak et al. (55) might be explained by the short number of treatment sessions or by the effects of contingent abstinence. While measurement of cravings is clinically useful and may point to mechanisms through which neurostimulation works (e.g., regulation of reward pathways), they represent subjective ratings of an introspective phenomenon (61) and therefore are subject to bias.

Unverified self-reported changes in consumption were present in five of the reviewed studies. Although this is more informative regarding effectiveness, biologically-verified measures of consumption represent a more objective measure of changes in substance use and should be employed in future investigations. Moreover, immediately before and after stimulation, fMRI and EEG measures of addiction-related circuitries would be helpful in assessing changes produced through stimulation (62).

Cognitive outcomes were reported in four of the reviewed studies. Improvements were found in three, which may be explained through direct effects of stimulation on targeted brain regions (e.g., DLPFC) mediating cognitive performance or indirectly through reduced substance use. Of note, previous studies have found support for nicotine-induced improvements in SCZ cognitive impairments, specifically in attention, visuospatial working memory, and verbal learning and memory; it has been proposed that these factors may contribute to increased tobacco addiction vulnerability in people with SCZ (47, 63–66). It is also possible that alleviation of clinical symptoms as a direct result of the neuromodulation or an indirect result of reduced substance use may have contributed to improvements in cognitive functioning due to reduced cognitive load or enhancement of cognitive resources. While conclusions are limited due to the preliminary nature of the evidence, future research should investigate whether neuromodulation interventions in prodromal SCZ aimed at improving cognitive deficits are effective in reducing the likelihood of future tobacco or cannabis use disorder.

Evidence of alleviation of depression or positive and negative SCZ symptoms was mixed. However, given that neuromodulation has also been used to ameliorate positive and negative symptoms (67, 68) and meta-analyses have shown rTMS to be effective in the treatment of both major depression and schizophrenia (69). Thus, future studies should continue to investigate the possibility of neuromodulation as an integrated treatment, as well as potential pathways to efficacy via reductions in negative symptoms, while controlling for symptom changes that are associated with reductions in substance use.

While this review shows preliminary support for the use of neuromodulation in individuals with SCZ and SCA, there remains a gap in evidence supporting its use in other psychotic disorders (e.g., bipolar disorder with psychotic features, first-episode psychosis). To that end, there remains a question of who the appropriate candidate for brain stimulation is; would individuals with acute substance-related exogenous psychosis (70) or first-episode psychosis (71) benefit from neuromodulation or should it be reserved for individuals experiencing more chronic and resistant psychosis? Additionally, case-studies (72–74) of rTMS in individuals with mood disorders have reported the occurrence of neuromdulation-induced mania as an adverse event, which is particularly relevant to treating SUDs in individuals with bipolar or depressive disorders with psychotic features. It is emphasized that caution should be exercised and that further empirical research should be conducted to establish definitive guidelines for clinicians.

### Limitations

There are a number of limitations to the current review. Primarily, is that despite the high prevalence of cannabis, stimulant, alcohol, and polysubstance use in SCZ and other psychotic-spectrum disorders (75–77), gaps remain in elucidating the effectiveness of neurostimulation for these substances, with only one study investigating cannabis in this population to date (56). Future studies of neuromodulation in SCZ should examine these substances for a more comprehensive understanding of its utility in treating SUDs. Furthermore, despite similar patient samples and outcome measures across studies, the stimulation parameters and targeted brain regions were highly heterogenous. In addition, self-reported substance use is subject to recall bias (78). Future studies should aim to biologically verify reductions in self-reported substance use. Moreover, antipsychotic medications that antagonize D2 dopamine receptors are known to reduce the effectiveness of tDCS on psychopathological symptoms (79), and this was not factored into the included studies. Participants in Smith et al. (59) and Brunelin et al. (52) were on clozapine during stimulation treatment, potentially impacting results. There were no studies of DBS in psychosis-spectrum populations, however, the feasibility of recruiting such patients for invasive brain stimulation procedures may prove challenging. Finally, the majority of the studies were conducted with predominantly male samples. While this may in part be due to sex differences in the diagnosis of SCZ (80) it limits generalizability of these results to females with SCZ.

## Conclusions and Future Directions

rTMS is a promising and well-tolerated option in the treatment of tobacco, and possibly, cannabis use disorders in SCZ and SCA. However, there is a need to optimize stimulation parameters (e.g., frequency, duration, and stimulation target regions), as has been noted in previous reviews (18). In addition, while this review suggests 5-10 sessions of tDCS may not be effective for reducing tobacco use in SCZ, future research should investigate whether more sessions may have efficacy. Larger sham-controlled clinical trials with longer follow-ups and more accurate substance use measures are needed to establish the efficacy of neuromodulation in reducing drug consumption, ideally in comparison to existing pharmacological and behavioral interventions. Moreover, future research should investigate the effects of rTMS on consumption of alcohol and other drugs (e.g., cannabis, cocaine, methamphetamine, opioids) in SCZ and other psychosis-spectrum illness.

## Data Availability Statement

The original contributions presented in the study are included in the article/supplementary material, further inquiries can be directed to the corresponding author/s.

## Author Contributions

SJ wrote the manuscript. SJ, MS, and NA-S conducted the systematic review. VS, GP, MS, DL, DC, and TG edited and revised the manuscript. TG, DC, SJ, and MS conceptualized the review. All authors contributed to the article and approved the submitted version.

## Funding

This work was funded in part by NIH grant R21-DA-043949 to TG.

## Conflict of Interest

The authors declare that the research was conducted in the absence of any commercial or financial relationships that could be construed as a potential conflict of interest.

## Publisher's Note

All claims expressed in this article are solely those of the authors and do not necessarily represent those of their affiliated organizations, or those of the publisher, the editors and the reviewers. Any product that may be evaluated in this article, or claim that may be made by its manufacturer, is not guaranteed or endorsed by the publisher.
